# Thermodynamics of the Interaction between Alzheimer's Disease Related Tau Protein and DNA

**DOI:** 10.1371/journal.pone.0104690

**Published:** 2014-08-15

**Authors:** Sergio Camero, María J. Benítez, Raquel Cuadros, Félix Hernández, Jesús Ávila, Juan S. Jiménez

**Affiliations:** 1 Departamento de Química Física Aplicada, Universidad Autónoma de Madrid, Madrid, Spain; 2 Centro de Biología Molecular Severo Ochoa, Consejo Superior de Investigaciones Científicas-Universidad Autónoma de Madrid, (CSIC/UAM), Madrid, Spain; 3 Centro de Investigación Biomédica en Red de Enfermedades Neurodegenerativas (CIBERNED), Madrid, Spain; Centre Hospitalier de l'Université Laval, Canada

## Abstract

Tau hyperphosphorylation can be considered as one of the hallmarks of Alzheimer's disease and other tauophaties. Besides its well-known role as a microtubule associated protein, Tau displays a key function as a protector of genomic integrity in stress situations. Phosphorylation has been proven to regulate multiple processes including nuclear translocation of Tau. In this contribution, we are addressing the physicochemical nature of DNA-Tau interaction including the plausible influence of phosphorylation. By means of surface plasmon resonance (SPR) we measured the equilibrium constant and the free energy, enthalpy and entropy changes associated to the Tau-DNA complex formation. Our results show that unphosphorylated Tau binding to DNA is reversible. This fact is in agreement with the protective role attributed to nuclear Tau, which stops binding to DNA once the insult is over. According to our thermodynamic data, oscillations in the concentration of dephosphorylated Tau available to DNA must be the variable determining the extent of Tau binding and DNA protection. In addition, thermodynamics of the interaction suggest that hydrophobicity must represent an important contribution to the stability of the Tau-DNA complex. SPR results together with those from Tau expression in HEK cells show that phosphorylation induces changes in Tau protein which prevent it from binding to DNA. The phosphorylation-dependent regulation of DNA binding is analogous to the Tau-microtubules binding inhibition induced by phosphorylation. Our results suggest that hydrophobicity may control Tau location and DNA interaction and that impairment of this Tau-DNA interaction, due to Tau hyperphosphorylation, could contribute to Alzheimer's pathogenesis.

## Introduction

Tau is a microtubule associated protein. It participates in the microtubule stabilization and organization system which regulates cellular morphogenesis, cytoskeleton functionality and axonal transport [1]–[6]. Alternative splicing gives rise to six isoforms expressed from the same gene in the CNS [7]. Tau protein contains a large number of serine and threonine phosphorylation sites. Hyperphosphorylation can be considered as one of the hallmarks of Alzheimer's diseases and other tauophaties [1], [8]–[11]. Together with the extracellular senile plaques, the intracellular tangles composed mainly of Tau protein, forming the paired helical filaments (PHFs), are the second type of aberrant proteinaceous aggregates found associated to Alzheimer's disease [12]–[15]. Tau is a highly soluble protein devoid of any well-defined secondary or tertiary structure, as many other proteins prone to aggregation and fibrillization also involved in neurodegenerative diseases. A survey of Tau literature leads one to conclude that aggregation and hyperphosphorylation must have a particular role in the neurodegenerative processes [16], [17]. However, a precise knowledge of those particular molecular events involving Tau protein in Alzheimer's disease yet remains elusive.

The interaction of Tau protein with DNA *in vitro* [18]–[23] and *in situ*, in neuronal [24] and non-neuronal [25] cells has been repeatedly reported. Although this protein is mainly found in the cytosol of neuronal cells, it has also been localized within the nucleus of neuronal [24], [26]–[31] and non-neuronal cells [25], [32]–[34]. Tau protein has also been found localized at ribosomes and plasma membrane of neuronal cells [35], [36]. The post-translational modification of the different isoforms of Tau together with its complex distribution in different cellular locations suggests a multifunctional role for this protein. Results concerning the Tau-microtubule interaction suggest that phosphorylation is involved in the regulation of Tau function. It was long time ago reported that phosphorylation produces a diminution in the affinity of Tau for the microtubule lattice [37], [38]. More recently, Tau phosphorylation by GSK3 has been reported to affect its axonal transport [39], [40]. Many other Tau functions such as interneuronal signaling, mitochondrial mobility or interaction with synaptic proteins have also been reported to be affected by phosphorylation (see reference [41] for review). Therefore we would say that Tau phosphorylation seems to be the general mechanism by which Tau function is regulated.

As for the nuclear location, studies on non-neuronal cells confer Tau an important role in nucleolar structure conformation and heterochromatinization of ribosomal genes [25], [34]. The role that it might play in the nucleus of neurons remains, however, to be elucidated, as well as the plausible physiological consequences derived from its interaction with DNA. A function related to protection of genomic integrity has been recently assigned to Tau protein [24]. These authors report that, similarly to the reversible nuclear translocation associated to dephosphorylation of many transcription factors (see [42] and references inside), oxidative or heat stress induce accumulation of dephosphorylated Tau protein within the nuclei of neurons. In addition, that nuclear translocation of Tau correlates with an increase of Tau-DNA interaction and DNA protection from heat stress damage [24]. In agreement with this observation, a recent report from our laboratory describes how Tau confers thermodynamic stability properties to DNA which are similar to those provided by histone, as deduced from *in vitro* experiments [43]. Similarly to the Tau-microtubule interaction, nuclear translocation of Tau is also regulated by phosphorylation, as many other Tau functions. However, the influence that phosphrylation may exert on the Tau-DNA interaction still remains unclear. Hua and He reported that phosphorylation of Tau with cdc2-like kinases did not affect the interaction with DNA [44]. A recent publication of Lu et al, however, describes how phosphorylation prevents Tau from DNA binding [45].

It seems that Tau translocation and the subsequent DNA binding and protection is reversible. It means that Tau binding to DNA must be controlled by the concentration inside the nucleus. The results we present here prove that, similarly to the Tau-microtubules interaction, Tau-DNA interaction is indeed dependent on the phosphorylation state of the protein, as shown by surface plasmon resonance and Tau expression in human embryonic kidney 293 (HEK) cells. Unphosphorylated, weakly aggregated forms of Tau, bind DNA reversibly, strengthening the concept of a functional regulatory role for Tau protein. The results of this reversible interaction characterized by means of surface plasmon resonance, allowed us to measure ΔG, ΔH and ΔS values, which suggest hydrophobicity must represent an important contribution to the Tau-DNA stability complex. Hyperphosphorylation of Tau could impair this interaction, therefore contributing to the Alzheimer's pathogenesis.

## Results

### Tau expression in HEK cells gives rise to phosphorylation in proliferating cells

**Figure 1:**
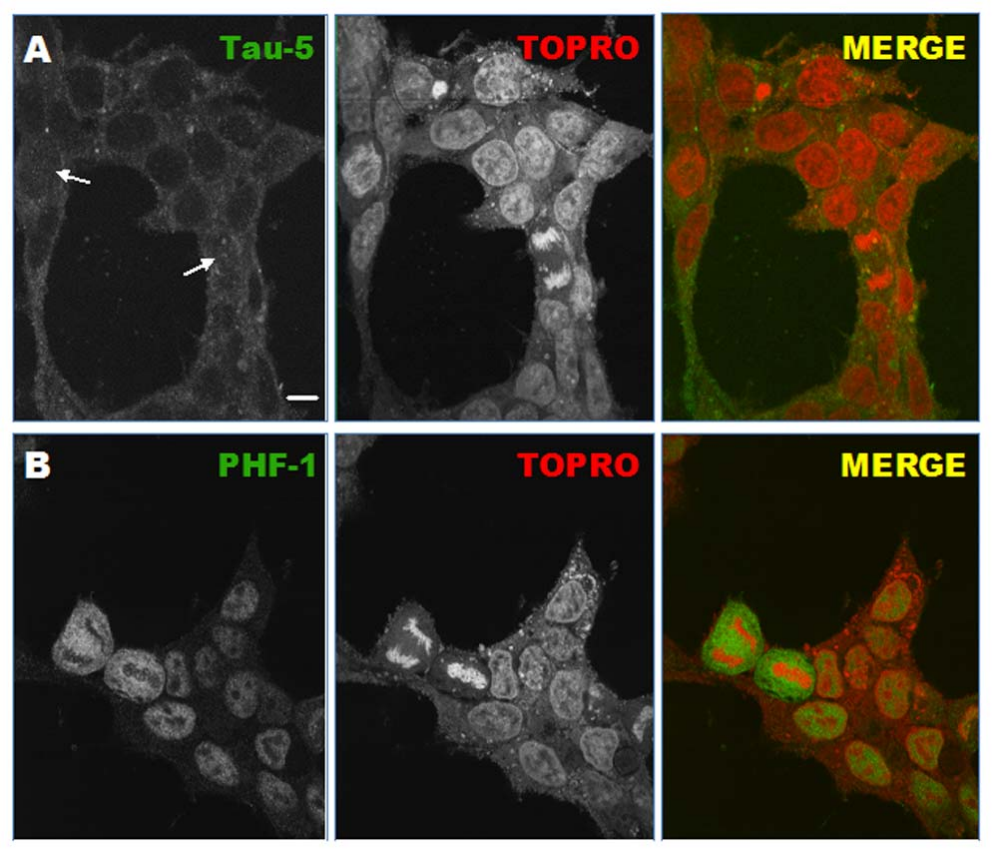
Confocal micrographs of Tau-stably expressed HEK-293 cells immunostained using PHF-1 and Tau-5 antibodies. Left column (green in grey scale) corresponds to Tau-5 (A, total Tau) and PHF-1 (B, phosphorylated Tau). Central column (red in grey scale) shows To-Pro-3 labeling (DNA). Right column shows merge images. Scale bar is equal to 10 µm.

Figure 1 shows the DNA and Tau localization in human embryonic kidney (HEK) cells expressing Tau protein in a stable form. Immunofluorescent confocal microscopy was used to study the distribution of both molecules in different populations of these cells. Staining with Tau5, an antibody which recognizes both phosphorylated and unphosphorylated forms of the protein, shows that Tau is mainly located in the cytosol of non-dividing cells (Fig. 1A). Nevertheless, Tau is also present inside the nuclei, although it seems to represent a minor proportion of total Tau. On the other hand, there are some To-Pro-3-labeled cells that have been pictured in the middle of division, displaying a highly condensed DNA (see Fig. 1A, TOPRO). In these cases where nuclear membrane disappears, certain proportion of labeled Tau protein (see arrows within Fig. 1A) share the same regions occupied by chromosomes, suggesting that a fraction of Tau colocalizes with DNA at some stages of cell division. A distinction between DNA (red in gray scale) and Tau (green in gray scale) starts to show up, still indicating that a fraction of Tau does not colocalize with DNA.

In Fig. 1B, PHF-1 antibody recognizes Tau molecules which are phosphorylated in serine 396 and 404. It can be observed that, as chromosomes starts to condensate in replicating cells, there is a clear distinction between DNA (red in gray scale) and phosphorylated Tau (green in gray scale). Interestingly, PHF-1 signal increases when the division process is started, what means that most of Tau recognized by Tau5 antibody must be progressively phosphorylated from the onset of cell cycle by nuclear kinases. The fact that phosphorylated Tau does not colocalize with condensed chromosomes in those situations where nuclear membrane disappears, suggests that phosphorylation would play a putative role preventing Tau-DNA interaction in replicating cells, similarly to the previously described microtubule binding inhibition induced by phosphorylation [37],[38].

### Effect of phosphorylation on the Tau-DNA interaction *in vitro*

**Figure 2:**
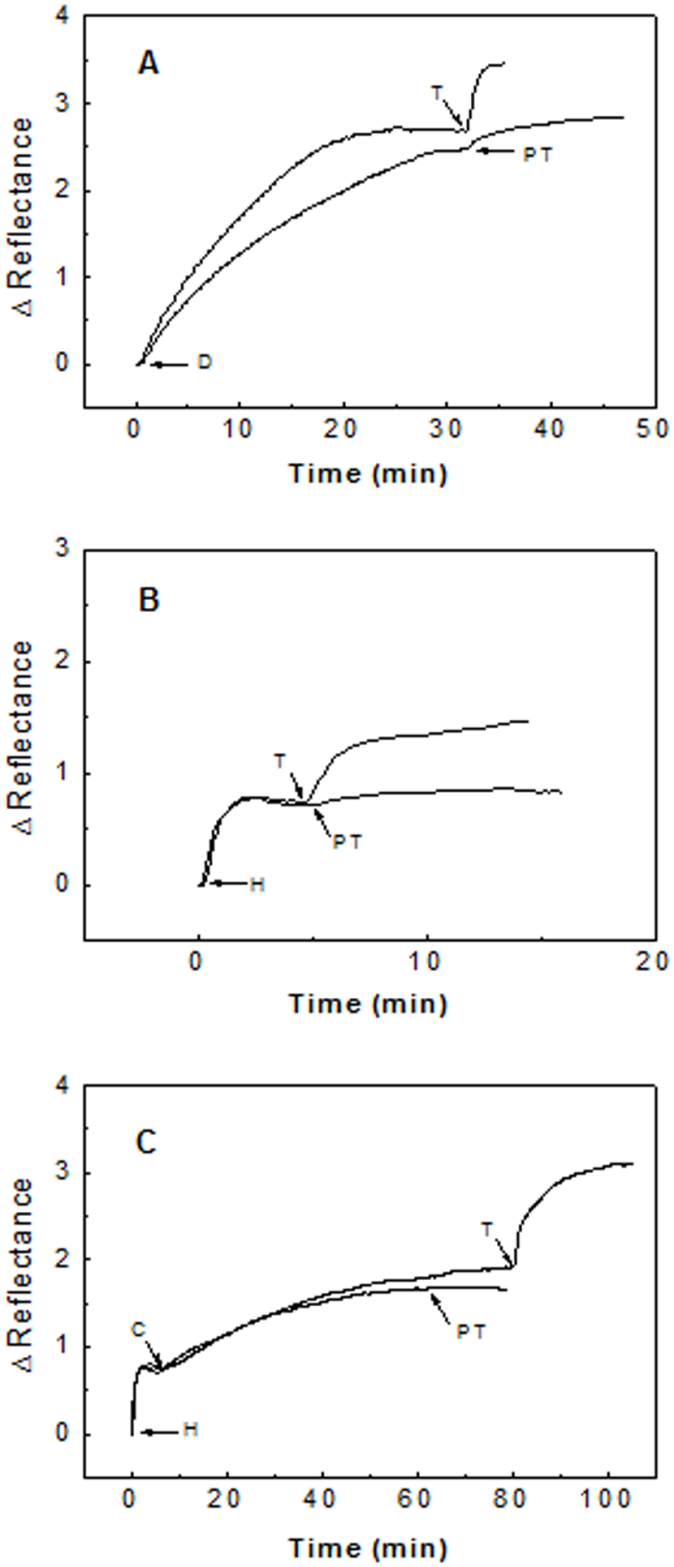
Effect of phosphorylation on the DNA-Tau protein interaction. **Part A:** Tau protein (12 µg/mL) (T) and phosphorylated Tau (12 µg/mL) (PT) binding to a DNA activated sensor surface (D). Part B: Tau protein (10 µg/mL) (T) and phosphorylated Tau (10 µg/mL) (PT) binding to the heparin activated sensor surface (H). Part C: Tau protein and phosphorylated Tau binding to a DNA-histone mixture immobilized on the sensor surface. DNA (8 µg/mL) was incubated with histone (8 µg/mL) for one hour. At the time indicated by C, the incubated mixture was flowed on a heparin activated surface. Tau protein (T) and phosphorylated Tau (PT), both at 10 µg/mL, were then flowed through the sensor cell. All phosphorylated Tau forms were flowed during at least 10 minutes, which is enough for the full binding of Tau protein in Figures 2A and 2B, and for more than 60% in Figure 2C.

Tau-DNA interaction *in vitro* was studied by flowing Tau solutions on a DNA activated sensor surface assembled on a surface plasmon resonance instrument. As it can be seen in part A of Figure 2, the unphosphorylated form of Tau binds DNA, as we have previously reported [43]. The reflectance-increase observed after injecting the Tau solution denotes the DNA-binding of the unphosphorylated form of Tau, purified from expression in E.coli.

Phosphorylated Tau-42 expressed in Sf9 cells only shows a small interaction with DNA, as can be seen in part A of Figure 2. The negative charge supplied by the phosphate groups are plausibly the cause of this loss of DNA binding capability showed by the phosphorylated form of Tau. Part B of the same Figure bears out it. As compared to the unphosphorylated form of Tau, the phosphorylated form also shows a small interaction with heparin. Basic amino acids of phosphorylated Tau form might be responsible of these residual interactions with heparin and DNA. Part C of the same Figure supports this. A mixture composed of DNA and histone was immobilized on the sensor surface. Unphosphorylated form of Tau is readily immobilized on this surface. However, the phosphorylated form does not bind this histone-DNA complex. The previously shown residual binding of phosphorylated Tau to DNA disappeared. These results suggest that Tau may be phosphorylated on those sites closely related to the interaction with DNA.

The loss of interaction with DNA induced by Tau phosphorylation does not seem to be related to dramatic changes in the aggregation state of the protein. The UV spectrum does not show the acute light scattering profile usually found for large aggregating protein forms (see Figures S1 and S2 in File S1). Besides that, phosphorylated Tau could be bound to polylysine activated surfaces, displaying a layer thickness slightly larger than that for the unphosphorylated form (Figure S3 in File S1), which indicates that oligomers within the range of 1 to 2 nm can be formed but no large fibrillar aggregates.

### The thermodynamics of the unphosphorylated Tau-DNA interaction, as probed by Surface Plasmon Resonance

**Figure 3:**
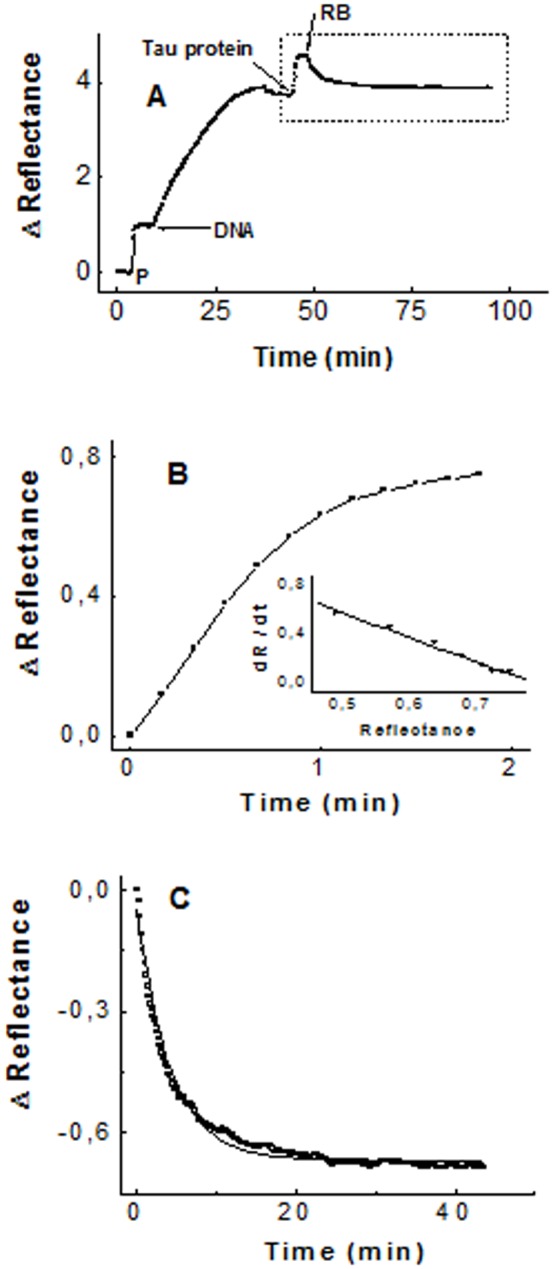
Reversible binding of Tau protein to DNA immobilized on the sensor surface. Part A: Reflectance time-course of the interaction. Solutions were injected at the time indicated by arrows: 8 µg/mL DNA and18 µg/mL Tau protein. RB stands for running buffer. Parts B and C show the kinetic analysis of the association and dissociation processes respectively.

**Figure 4:**
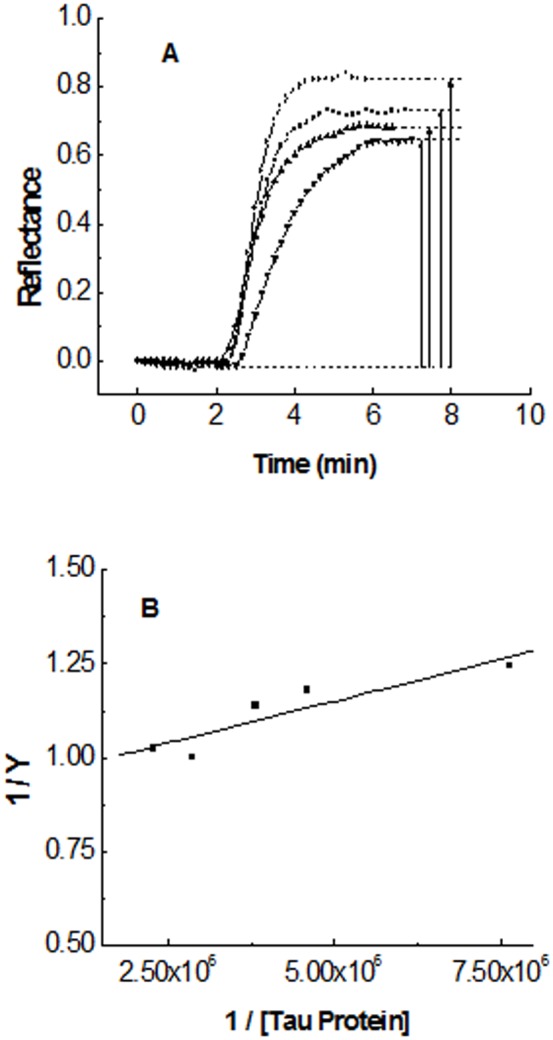
Equilibrium binding of Tau protein to DNA immobilized on the sensor surface. Part A: Reflectance time-course after flowing Tau protein solutions at different concentrations in µg/mL: 16 (•), 12 (▪), 10 (▴), 6 (▾). Part B: inverse plot of the concentration dependency of the fractional saturation (equation [10]).

As shown in part A of Figure 3, the reflectance-increase value derived from the Tau-DNA interaction results to be close to that reflectance-increase value corresponding to the last polylysine layer of the PHP structure routinely used in the sensor surface activation. This means that, according to the reported thickness value of the polylysine layer [46], the reflectance-increase observed when Tau is flowed on DNA must correspond to the building of a Tau layer on DNA, mainly composed of unfolded monomers or small Tau oligomers about 1 nm thick. Tau binding to DNA is reversible. Part A of Figure 3 shows how the binding of Tau to DNA is followed by the dissociation of the complex, when running buffer is injected to flow through the sensor cell. The detailed time dependence of complex formation as well as the complex dissociation are shown in parts B and C of the same Figure. Analysis of the dissociation process following a first order kinetics yields the first-order constant k-1. Analysis of the complex formation following a pseudo-first order kinetics, as described under Materials and Methods, yields then the second order rate constant, k_1_ (inset of part B). Quotient of constants, k_1_/k_-1_, renders the equilibrium constant for the Tau-DNA complex formation, K_eq_  =  (2.4 ± 0.4) 10^7^ M^−1^ (± SEM), obtained as the average from six determinations between 18 °C and 22 °C. This equilibrium constant is calculated under the assumption of the simple mechanism of interaction described in Materials and Methods for the Tau-DNA complex formation. As can be observed in part A of Figure 4, increasing concentration values of Tau in those solutions flowing through the sensor cell give rise to increasing surface saturation. It means that the DNA-activated sensor surface is saturated by running Tau, in a concentration dependent manner, due to the reversibility of the interaction. The saturation fraction, Y, can then be obtained from equation (11) as a function of the Tau protein concentration (equation 10). An inverse plot of this hyperbolic equation renders a straight line from which the Equilibrium Constant for the complex formation, K_eq_  =  (2.3 ± 0.6) 10^7^ is obtained.

**Figure 5:**
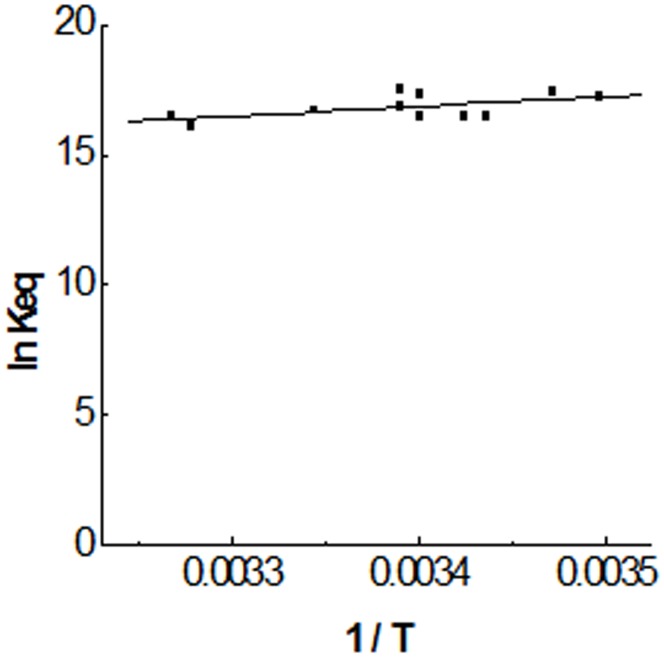
Van't Hoff plot: temperature dependence of equilibrium constant for the Tau-DNA interaction. Equilibrium constants values were obtained from the quotient k_1_/k_−1_, where k_1_ and k_−1_ are the rate constants obtained from the kinetic analysis (Table 1).

The Equilibrium Constant value calculated from equilibrium data does not depend on any kinetic assumption and agrees well with that value obtained previously from the kinetic model, K_eq_  =  (2.4 ± 0.4) 10^7^ M^−1^, therefore supporting the one-step mechanism of interaction proposed in the Materials and Methods section. The kinetic model used to obtain the rate constants for the complex formation and dissociation, k_1_ and k_−1_, was then used to study the dependency of Equilibrium Constants values on temperature, within the range of 13°C to 33 °C. The results, including those obtained between 18 °C and 22 °C are included in Table 1. It can be seen that Equilibrium Constant for the complex Tau-DNA barely changes within the selected range of temperature. A Vant'Hoff plot of the temperature dependency within this range (Figure 5) renders an enthalpy change for the complex formation, ΔH°  =  −32 ± 15 kJ mol^−1^. The potential energy decrease associated to Tau-DNA complex formation is given by ΔG°  =  − RT ln Keq  =  −41.4 ± 0.5 kJ mol^−1^ at 20 °C. Accordingly, the entropy change, following ΔS^o^  =  (ΔH^o^ − ΔG^o^)/T, results to be close to zero, ΔS°  =  0.03 ± 0.05 kJmol^−1^K^−1^. Tau protein lacks of a well-defined secondary or tertiary structure in solution. It means that Tau may adopt a huge number of possible conformations for the polypeptide chain, each of them housing, in turn, a huge number of quantum states. Therefore, immobilization of Tau on the DNA molecule would have to involve a decrease in the number of quantum states available to Tau, with the corresponding large decrease of entropy. The positive, almost null value for the entropy variation obtained suggests that, together with the large diminution of entropy derived from the Tau immobilization on DNA, there must be a compensating large increase in the number of quantum states accompanying the Tau-DNA complex formation. This entropy increase may have its origin in the release of water molecules which would confer a strongly hydrophobic character upon the Tau-DNA complex formation.

**Table 1:** Kinetic constants for the Tau-DNA complex formation (k_1_) and complex dissociation (k_−1_) and the corresponding equilibrium constants, obtained as k_1_/k_−1_, at different temperatures.

Temperature (°C)	k_−1_ (min^−1^)	k_1_ × 10^−6^ (M^−1^min^−1^)	Keq × 10^−7^ (M^−1^)
13	0.17	5	2.9
15	0.13	5	3.8
18	0.27	4	1.5
19	0.22	3.3	1.5
21	0.36	5.3	1.5
21	0.14	4.8	3.4
22	0.24	5.2	2.2
22	0.15	6.1	4.1
26	0.4	7.4	1.8
32	0.32	3.2	1
33	0.33	4.9	1.4

## Discussion

We describe here the interaction of unphosphorylated Tau with calf thymus DNA, immobilized on a sensor surface. It has been shown to be reversible and, according to the reflectance value increase, mostly attributable to monomeric or small oligomeric forms of the protein. Surface plasmon resonance supplies interaction data in real time. It allows therefore the quantitative analysis of the interaction from a kinetic point of view. The second order rate constant for the association of Tau with DNA, k_1_, and the first-order rate constant for the complex dissociation, k_−1_, can be obtained from the time-dependency analysis of the reflectance changes registered. Equilibrium constant for the complex formation, K_eq_  =  k_1_/k _−1_ M^−1^, can then be calculated on the assumption of the simple mechanism described in Materials and Methods. On the other hand, the reversibility of the interaction offers the possibility of obtaining the equilibrium constant from data concerning the equilibrium fractional saturation, independently of any assumption on the interaction mechanism. Both equilibrium constants values, the one obtained from kinetic data and that from equilibrium saturation are in good agreement, therefore confirming the simple one-step mechanism of interaction, as well as the absence of any kind of cooperativity within the experimental error of our measurements. The dissociation constant value obtained, Kd  =  0.04 µM, means that Tau binds DNA with a comparable affinity to that shown for the binding to microtubules, Kd  =  0.1 µM as reported by Ackmann et al [47]. A reversible binding of Tau to DNA has also been described by Krylova et al [22], as obtained from a non-equilibrium capillary electrophoresis method. The dissociation equilibrium constant reported by these authors is between 0.3 and 0.9 µM. This different value may be due to the fact that we measure the binding of Tau to an immobilized DNA sample, or to the different size of the molecule selected as a source on DNA: small oligonucleotides in the experiments of Krylova et al, in contrast to calf thymus DNA used in our measurements. Of course, both different experimental conditions might contribute to the different equilibrium constants obtained.

The neuronal concentration of Tau is about 2 µM [48]. According to the Kd values reported here for the DNA interaction with Tau, this concentration would be high enough to saturate reversibly the potential DNA-sites available *in vivo*. The activity of different types of kinases might additionally affect Tau residues involved in DNA binding. Sf9 cells can be considered as a good model to simulate the phosphorylation extent of Tau. Tau phosphorylation sites associated to Alzheimer's disease have been classified into two categories, the KXGS and the SP/TP motifs [49]. Phosphorylation profile of Tau in transfected Sf9 cells has been shown to be similar to that of neuronal cells [38]. Our results show that phosphorylation due to endogenous kinases of Sf9 cells somehow induces changes in Tau which alter Tau binding to DNA, in agreement with the lack of colocalization with condensed chromosomes shown in Figure 1 above. Therefore, Tau phosphorylation might regulate the availability of unphosphorylated Tau to bind DNA in a reversible manner. It has been reported that heat stress induces Tau dephosphorylation and nuclear translocation of dephosphorylated Tau, concomitantly with an increase of Tau-DNA interaction and DNA protection. The full process is reversed when the insult ceases [24]. This observation is in agreement with our results showing that binding of dephosphorylated Tau to DNA is reversible. According to our thermodynamic data, changes in the concentration of dephosphorylated Tau available to DNA must be the variable determining the extent of Tau binding and DNA protection. This phosphorylation-dependent regulation of DNA binding is reminiscent of that reported for the Tau-microtubule interaction regulated also by Tau phosphorylation [41], [48].

Tau is an intrinsically disordered protein containing 85 phosphorylatable sites, including 45 serines, 35 threonines and 5 tyrosines [50]. It means that the number of differently phosphorylated forms of the protein (2^85^) by far exceeds the availability of Tau molecules not only within a neuron but even within the full brain, therefore suggesting that phosphorylation might form part of a subtle mechanism dedicated to modulate protein location and protein-protein interactions rather than to generate differently phosphorylated forms having specific properties. Molecular dynamics calculations have shown how phosphorylation may induce changes in protein surface hydrophobicity which may be a determinant of localization to interact with different partners within the cell [51]. Indeed Tau, like many proteins lacking of a well defined structure may interact with a large number of molecular structures. More than thirty partners have been described [52], including different proteins, DNA and of course microtubules. It results conceivable that such extended phosphorylation range offered by Tau may be dedicated to supply a fine modulation of the molecular surface hydrophobicity required to regulate the Tau location and partnership variability. We have found that in fact, hydrophobicity must play an important role in Tau-DNA interaction. Tau protein may adopt a huge number of conformations in solution, as a consequence of the lack of well-defined secondary or tertiary structure. Therefore, one would expect that Tau immobilization on DNA were associated to a large loss of entropy, *i.e.* a large and negative value of ΔS. The small and positive value found for ΔS suggests that a large increase of entropy must parallel the entropy lost derived from the Tau immobilization on DNA. The release of water molecules as a consequence of the DNA interaction, with the consequent availability of translational energy levels, might produce that entropy increase. This positive hydrophobic contribution to the entropy change would compensate the negative contribution associated to the Tau immobilization. The result is a small value for the change of entropy, ΔS, and, according to the relationship ΔH  =  ΔG + TΔS, a small value of enthalpy, ΔH, and the consequent small influence of temperature on the equilibrium constant, according to the Van't Hoff equation. This hypothetical hydrophobic contribution to the Tau-DNA interaction would be in agreement with the role that hydrophobicity has been reported to play in the binding of Tau to microtubules, as well as in the Tau-Tau interactions leading to the paired helical filaments formation. Electrophoretic mobility assays of DNA in the presence of truncated forms of Tau show that the microtubule binding domain (MTBD) of Tau contributes to the DNA interaction [23]. The MTBD has also been involved in the self-assembling of Tau [53]–[55]. Although Tau protein has a low content of hydrophobic residues, two hydrophobic stretches can be found at the beginning of the R2 and R3 repetitions forming part of the MTBD, and hydrophobic interactions have been proposed to play a major role in Tau-Tau interaction and fibrils formation [56], [57].

Tau spontaneously forms small oligomers, protofibrils and fibrillar fragments [58]. Reversibility of Tau-DNA interaction was lost two months after purification. The binding was then mostly irreversible, concomitantly with the process of aggregation. Under these conditions Tau would remain irreversibly bound to DNA in a way that is independent of heat/oxidative insults. On the other hand, massive phosphorylation of Tau, besides depleting the available normal tau in the neuron by sequestering it [59], would prevent it from nuclear translocation and the consequent DNA protection. Protein aggregation and massive phosphorylation are the two main hallmarks found in Tau protein linked to tauophaties, among which the most known is Alzheimer's disease. If not a coincidence, one would conclude that both Tau modifications might contribute to the onset of these neurological disorders by impairing the reversible Tau-DNA interaction and leading to a Tau-induced DNA deregulation.

## Materials and Methods

### Chemicals/Reagents

DNA from calf thymus (activated type XV), unfractionated whole histone from calf thymus (type II-A), poly-L-Lysine, heparin and hepes were from Sigma (St. Louis, MO, USA). SPR gold sensorchips were from Xantec Bioanalytics (Dusseldorf, Germany). PHF-1 antibody was kindly donated by Dr. Davies (Albert Einstein College of Medicine, NY, USA). According to residue numbering of the longest human Tau isoform (441 aa), PHF-1 antibody react with Tau when serines 396 and 404 are phosphorylated. Total tau protein was detected with Tau-5 antibody from Abcam (Cambridge, MA, USA). Unless otherwise indicated, all experiments were carried out in a neutral running buffer composed of 10 mM Hepes, 0.1 M NaCl, pH 7.

### Cell culture HEK 293 Tau cells and immunofluorescence analysis

HEK 293 Tau expressing cells (expressing Tau 3R isoform, a kind gift from Dr. Miguel Medina) were grown in Dulbecco's modified Eagle's medium (DMEM) supplemented with 10% fetal bovine serum, 2 mMGlutamine, 1 mM Piruvate, 100 U/mL penicillin, 100 U/mL streptomycin and 0.2 mg/mL Zeocin in a humidified atmosphere of 5% CO_2_/95% air at 37 °C. Proliferating HEK 293 Tau expressing cells were plated on glass coverslips coated with 1 mg/mL poly-L-lysine, maintained in the same culture medium for 24 hours. For immunofluorescence analysis HEK cells were fixed with 4% paraformaldehyde for 20 min at 37 °C and then washed with PBS. Fixed cells were incubated with 1 M glycine for 30 min and then permeabilized with 0.2% Triton X-100 in PBS for 5 min at room temperature. The coverslips were blocked with 1% BSA/PBS for 1 hour at room temperature and incubated in primary antibodies (PHF-1 or Tau-5 1/100) in 1% BSA, in PBS, for 1 hour at room temperature. After washing 3 times with PBS, the secondary antibodies were incubated for 1 hour at room temperature. To-pro-3 1µM (Invitrogen, Carlsbad, CA, USA) staining to label nuclei was performed 10 minutes before finishing secondary antibody incubation. Finally, the coverslip were washed 3 times with PBS and once with H_2_O, and mounted with FluorSave Reagent (Calbiochem, La Jolla, CA, USA). Fluorescence microscopy was used to measure fluorescence intensity. After staining with PHF-1 antibody, cells were observed on a Zeiss Axiovert 200 fluorescent microscope (Carl Zeiss MicroImaging, Thornwood, NY, USA). PHF-1 antibody fluorescence images were captured through a 100 × lens on a high resolution CCD camera (SPOT-RT Sliders, Diagnostic Instrument Inc, MI, USA).

### Tau purification

The detailed purification procedure of the unphosphorylated longest human Tau isoform, (hTau 42) has been described in a previous contribution from this laboratory [43]. After expression in E.coli BL21 (DE3), Tau protein was purified from the supernatant resulting from bacteria sonication and centrifugation. The protocol used was based on that described by Lindwall and Cole [60], which makes use of the heat-and acid-stability of Tau, together with its insolubility in 25% of glycerol solutions. Tau was stored at −20 °C in a buffer composed of Hepes 10 mM, NaCl 100 mM and EDTA 1 mM, pH 7. All measurements were carried out after room-temperature incubation for the time period indicated for each experiment. The isolated protein was characterized by electrophoresis, UV-absorbance spectroscopy, and Western blot. Tau protein concentration was obtained by using an extinction coefficient ε_M_  =  7700M^−1^cm^−1^ at 280 nm [61].

### Expression and purification of phosphorylated Tau protein

Tau protein, phosphorylated on at least Ser 202, Thr 205 and Thr 212, Ser 214 as shown by antibodies AT8 and AT100, respectively [62], was obtained from an insect cell culture infected with baculoviruses expressing the longest human Tau isoform of 441 amino acids. Sf9 cells were grown at 27 °C in 35 mm dishes, in fetal bovine serum (Invitrogen, Carlsbad, USA) with antibiotics (penicillin and streptomycin). Monolayer cultures of Sf9 cells (3 × 10^7^/mL) were infected with baculoviruses encoding Tau-42 cDNA and then incubated for 72 h at 27 °C without CO_2_ from the time of infection. Infected cells were suspended in 1 mL of 0.1 M Tris, 100 mM NaCl, 1 mM EGTA, 0.5 mM MgCl_2_, 1 mM sodium orthovanadate, 2.5 mM sodium pyrophosphate, 10 mM glycerophosphate, 100 nM okadaic acid and 0.25 mM PMSF, pH 7.5, and sonicated 2 × 5 min on ice. The homogenate was centrifuged at 10000 g for 30 minutes and the supernatant boiled for 15 minutes. After centrifugation under the same conditions, 50% saturated ammonium sulphate was added to the supernatant and incubated overnight at 4°C to precipitate Tau protein. Pellet collected after centrifugation was suspended in 1 mL PBS and dialyzed against 0.1 M Mes, 2 mM EGTA, 0.5 mM MgCl_2_, pH 6.7. Purity of both forms of Tau, phosphorylated and unphosphorylated, was verified by electrophoresis (see Supporting information). UV absorbance spectra were obtained from both forms, showing maxima within the range of 270–280 nm (Figures S1 and S2 in File S1).

### Surface Plasmon Resonance

Plasmon resonance has been obtained by means of a homemade experimental set-up based on the proposals of Kretschmann and Raether [63], following the description made by Liedberg et al [64], as it has been described in previous reports [46], [65], [66]. This technique monitors macromolecular interactions taking place at the interface between a solid (usually gold) surface and a buffered solution. One of the two interacting partners has to be immobilized on the gold surface, while the second one flows through a cell, one wall of which is the metallic solid surface. A monochromatic laser radiation (632.8 nm) from a He-Ne laser is then directed upon the interface, under total reflection conditions. Interaction between the immobilized molecules and those flowing through the cell produces changes in the interface refraction index, which, in turn, provokes changes in the light reflected intensity, therefore supplying a physical signal to follow the interaction in real time.

The gold surface is covered by a three layer structure composed of two polylysine layers bound to each other by a polyanionic layer of heparin in the middle. The first polylysine layer is bound to gold, while the second one is exposed to the solvent [46]. This three layer structure houses the immobilized partner by interacting with the external polylysine layer, and prevents biological molecules from direct contact with gold. It is stable at neutral pH and is easily rebuilt by changing the pH value from neutral to basic, therefore allowing for consecutive experiments using the same experimental set-up for different interaction experiments.

The simplest mechanism describing the reversible interaction of Tau protein with DNA immobilized on the PHP activated gold surface (DNAi) is:



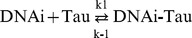
([1])


The differential equation defining both reversible steps is:



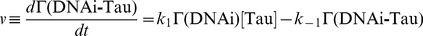
(2)


where [Tau] stands for the Tau protein concentration in the solution running through the flow cell. Γ(DNAi) and Γ(DNAi-Tau) stand for the surface concentration of immobilized DNA on the PHP activated surface and the DNA-Tau complex respectively. Following equation [1],



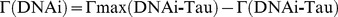
(3)


where Γmax(DNAi-Tau) stands for the maximal surface concentration of Tau bound to immobilized DNA. As mentioned above, the consequence of the Tau binding on DNA is an increase of reflectance, which will be proportional to the surface concentration of Tau bound to DNA (see reference [67] for details):



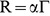
(4)
[4]



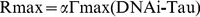
(5)


Substituting equations [3], [4] and [5] into the kinetic equation [2] yields:



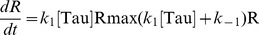
(6)


Tau protein is constantly supplied by the flow. Its concentration may, therefore, be considered constant. Consequently, under these pseudo-first order conditions, a plot of dR/dt versus R must be a straight line, the slope of which corresponds to (k1 [Tau] + k-1). The last expression yields the k1 value after knowing k-1. This constant was obtained from the first-order fitting of the dissociation complex as probed at real time by the decreasing reflectance measured when Tau protein solution was substituted by running buffer.

The equilibrium constant for the binding of Tau on the immobilized DNA comes defined by:



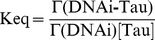
(7)


The saturation fraction, Y, of the sensor surface comes given by:



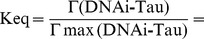
(8)
[8]

Solving equation [7] for Γ(DNAi-Tau) and substituting equation into [8] renders:



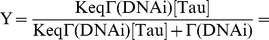
(9)
[9]

Rearranging,



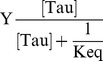
(10)


Equation [10] is the Langmuir-like hyperbolic equation describing the binding of a ligand, Tau, on a set of independent binding sites on the immobilized DNA.

The equilibrium values for Y can be easily obtained making use of equations [4] and [5]



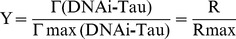
(11)


Unless otherwise indicated, values following the ± symbol represent the standard error of the data as obtained for a linear fitting.

## Supporting Information

File S1 **SPR immobilization upon polylyisine, SDS-PAGE and UV spectra of phosphorylated/unphosphorylated Tau protein.**
(DOC)
