# Supplementary material for: Thermodynamics of the Interaction between Alzheimer's Disease Related Tau Protein and DNA
Source: PLoS One. 2014 Aug 15;9(8):e104690. doi: 10.1371/journal.pone.0104690 (PMC4134230; doi:10.1371/journal.pone.0104690)
Supplement: File S1 — SPR immobilization upon polylyisine, SDS-PAGE and UV spectra of phosphorylated/unphosphorylated Tau protein. (DOC) [file pone.0104690.s001.doc]

Recombinant human Tau protein (hTau42) was purified from *E.coli* and Sf9 cells as described [43, 62]. The purity of these proteins was assessed by SDS-polyacrylamide gel electrophoresis and UV-spectroscopy (Figures S1 and S2).


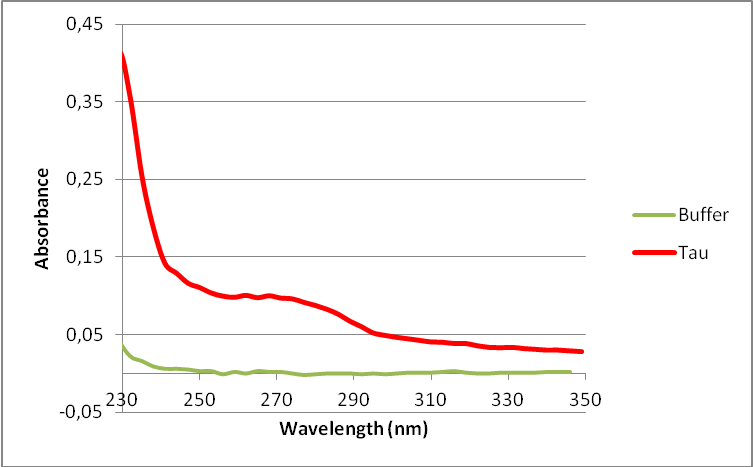

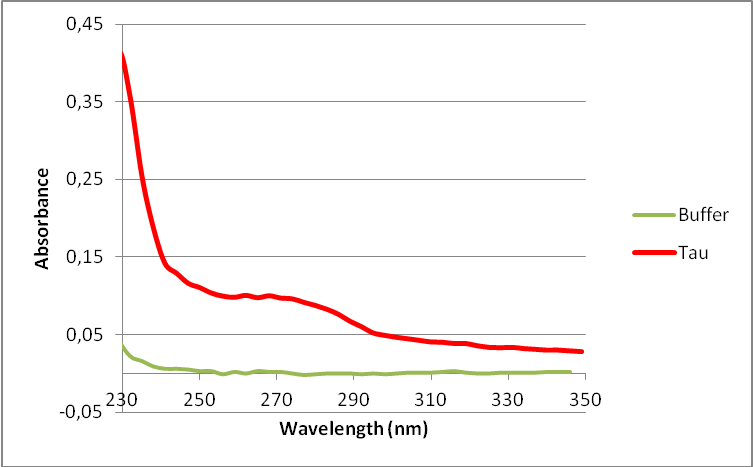

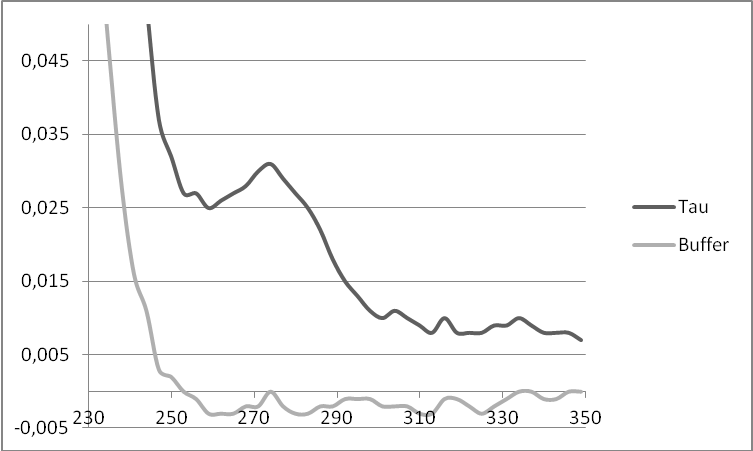


1

2


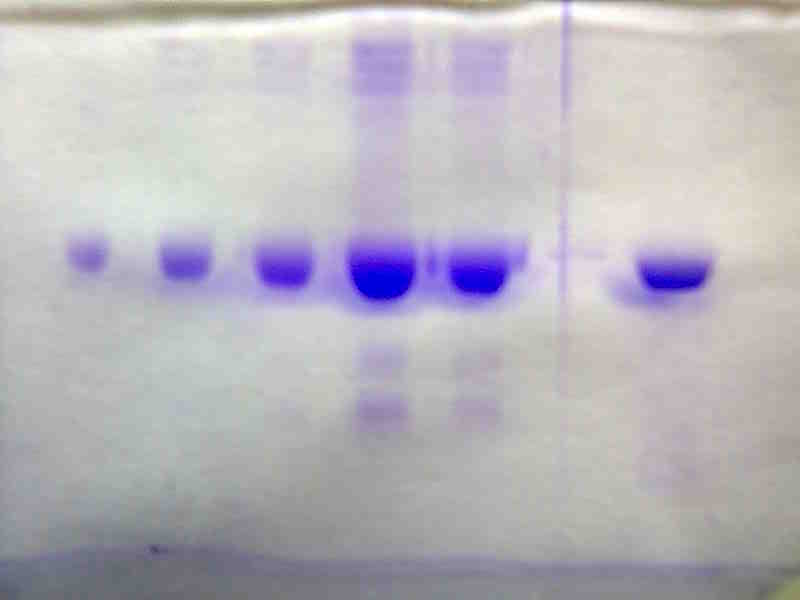


**Figure S1. Purity of Tau protein purified from *E.coli.*** Polyacrylamide gel electrophoresis: Lane 1: Bovine serum albumin (5μg). Lane 2: Tau protein (4 μL). UV spectroscopy: Black line: Tau protein. Grey Lane: Buffer.

1

2


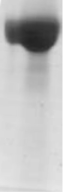

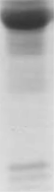


**Figure S2. Purity of Tau protein purified from Sf9 cells*.*** Polyacrylamide gel electrophoresis: Lane 1: Bovine serum albumin (10 μg). Lane 2: Phosphorylated Tau protein (10 μL). UV spectroscopy: Black line: Phosphorylated Tau protein. Grey lane: Buffer.

**Figure S3: Tau immobilization on polylysine.** Unphosphorylated Tau protein (10 μg/mL) (T) and phosphorylated Tau protein (10 μg/mL) (PT) bound to the polylysine activated sensor surface (P).
